# Event-Related Potentials in a Cued Go-NoGo Task Associated with Executive Functions in Adolescents with Autism Spectrum Disorder; A Case-Control Study

**DOI:** 10.3389/fnins.2017.00393

**Published:** 2017-07-11

**Authors:** Anne L. Høyland, Geir Øgrim, Stian Lydersen, Sigrun Hope, Morten Engstrøm, Tonje Torske, Terje Nærland, Ole A. Andreassen

**Affiliations:** ^1^Department of Mental Health, Faculty of Medicine and Health Sciences, Regional Centre for Child and Youth Mental Health and Child Welfare, Norwegian University of Science and Technology Trondheim, Norway; ^2^Department of Pediatrics, St. Olavs Hospital, Trondheim University Hospital Trondheim, Norway; ^3^Neuropsychiatric Unit, Østfold Hospital Trust Fredrikstad, Norway; ^4^Department of Psychology, Norwegian University of Science and Technology Trondheim, Norway; ^5^Norwegian Centre for Mental Disorders, KG Jebsen Centre for Psychosis Research, University of Oslo Oslo, Norway; ^6^Department of Neurohabilitation, Oslo University Hospital Oslo, Norway; ^7^Department of Neurology and Clinical Neurophysiology, St. Olavs Hospital, Trondheim University Hospital Trondheim, Norway; ^8^Department of Neuromedicine and Movement Science, Norwegian University of Science and Technology Trondheim, Norway; ^9^Division of Mental Health and Addiction, Vestre Viken Hospital Trust Drammen, Norway; ^10^NevSom, Department of Rare Disorders and Disabilities, Oslo University Hospital Oslo, Norway; ^11^Division of Mental Health and Addiction, Oslo University Hospital Oslo, Norway

**Keywords:** ASD, executive functions, Go-NoGo task, ERP, CNV, N2, P3, insistence of sameness

## Abstract

Executive functions are often affected in autism spectrum disorders (ASD). The underlying biology is however not well known. In the DSM-5, ASD is characterized by difficulties in two domains: Social Interaction and Repetitive and Restricted Behavior, RRB. Insistence of Sameness is part of RRB and has been reported related to executive functions. We aimed to identify differences between ASD and typically developing (TD) adolescents in Event Related Potentials (ERPs) associated with response preparation, conflict monitoring and response inhibition using a cued Go-NoGo paradigm. We also studied the effect of age and emotional content of paradigm related to these ERPs. We investigated 49 individuals with ASD and 49 TD aged 12–21 years, split into two groups below (young) and above (old) 16 years of age. ASD characteristics were quantified by the Social Communication Questionnaire (SCQ) and executive functions were assessed with the Behavior Rating Inventory of Executive Function (BRIEF), both parent-rated. Behavioral performance and ERPs were recorded during a cued visual Go-NoGo task which included neutral pictures (VCPT) and pictures of emotional faces (ECPT). The amplitudes of ERPs associated with response preparation, conflict monitoring, and response inhibition were analyzed. The ASD group showed markedly higher scores than TD in both SCQ and BRIEF. Behavioral data showed no case-control differences in either the VCPT or ECPT in the whole group. While there were no significant case-control differences in ERPs from the combined VCPT and ECPT in the whole sample, the Contingent Negative Variation (CNV) was significantly enhanced in the old ASD group (*p* = 0.017). When excluding ASD with comorbid ADHD we found a significantly increased N2 NoGo (*p* = 0.016) and N2-effect (*p* = 0.023) for the whole group. We found no case-control differences in the P3-components. Our findings suggest increased response preparation in adolescents with ASD older than 16 years and enhanced conflict monitoring in ASD without comorbid ADHD during a Go-NoGo task. The current findings may be related to Insistence of Sameness in ASD. The pathophysiological underpinnings of executive dysfunction should be further investigated to learn more about how this phenomenon is related to core characteristics of ASD.

## Introduction

Autism spectrum disorder (ASD) is a developmental disorder with impaired reciprocal interaction and a restricted pattern of behavior (ICD-10, 1992)/(DSM-5, 2013). Insistence of sameness was described as part of autism already by Kanner in 1943 who stated “anxiously obsessive desire for the maintenance of sameness” as part of the behaviors of the disorder (Kanner, 1943). Insistence of sameness and resistance to change are core features of the Restrictive and Repetitive Behavior, RRB, in DSM-5 and the two categories of ASD symptoms in DSM-5 may represent independent cognitive components and neural patterns (Happe and Frith, 2006; Mandy and Skuse, 2008; Brunsdon and Happé, 2014).

A potential cognitive process that may be related to RRB is executive functions, which are high-level cognitive processes that control goal-directed behavior and include abilities such as response inhibition, interference control, working memory, and set shifting (Friedman and Miyake, 2017). Executive functions are often affected in neurodevelopmental disorders such as ASD (Hill, 2004; Pugliese et al., 2014) and have been shown to have broad and significant implications for everyday life (Miyake and Friedman, 2012; Downes et al., 2017). The prefrontal cortex is regarded as the main brain region involved in executive functions (Friedman and Miyake, 2017), and prefrontal processes seem also to be involved in RRB (Mosconi et al., 2009; Agam et al., 2010). RRB may be subdivided in two separate categories, Repetitive Sensory Motor Action and Insistence on Sameness. The use of RRB subcategories, particularly Insistence of Sameness behaviors, can create more behaviorally homogeneous subgroups of children with ASD (Bishop et al., 2013).

Many studies have explored the relationship between RRB and executive functions (Lopez et al., 2005; Happé and Ronald, 2008; Boyd et al., 2009; Mosconi et al., 2009; Agam et al., 2010; Van Eylen et al., 2015). Deficient response inhibition and reduced inhibitory control are specifically suggested involved in the Insistence of Sameness category (Turner, 1997; Mosconi et al., 2009; Agam et al., 2010). Holmboe et al. (2010) described that siblings of children with ASD showed reduced selective inhibition due to difficulties in disengaging attention, referred to as “sticky fixation.” This concept may be related to Insistence of Sameness. A recent study reported reduced inhibitory control in a Go-NoGo task in adults with ASD (Uzefovsky et al., 2016) and found an association between this and autistic traits measured by the Autism Spectrum Questionnaire. Investigating the neurobiology of these deficits, may contribute to a better understanding of the RRB in ASD.

The conventional measurement of executive functions has been cognitive performance-based tests (Toplak et al., 2013). This involves structured tasks in quiet, calm, distraction-free environments which may not represent the real-life situation with multiple demands and unclear goals in an environment of disturbing stimuli. Thus, the ecological validity of such measures is debated (Anderson et al., 2002; Mahone et al., 2002; Kenworthy et al., 2008; Isquith et al., 2013; Toplak et al., 2013). A supplement to laboratory testing is rating scales of executive functions in everyday life. The Behavior Rating Inventory of Executive Function, BRIEF (Gioia et al., 2000), is a questionnaire developed to identify everyday executive function abilities. These tests are thought to capture different levels of executive functions and provide a more complete picture of executive functions in everyday life (Isquith et al., 2013; Toplak et al., 2013). The parent rating scales (BRIEF) are capturing other aspects of executive functions than conventional performance-based tests, shown by the low-to-moderate correlations between them (Silver, 2014).

Little is known about the underlying pathobiology of executive dysfunction in ASD. One fruitful approach to investigating the pathobiology related to executive functions is through electrophysiology. Event Related Potentials (ERPs) are cerebral generated electrical voltages recorded on the scalp in response to specific stimuli or responses (Luck, 2014). A Go-NoGo task elicits ERPs associated with response preparation, conflict monitoring, and response inhibition; processes important to establish efficient and goal directed behavior and thus executive functions (Jonkman, 2006). Several lines of evidence suggest ERP correlates to executive dysfunction in psychiatric and neurodevelopmental disorders (Johnstone et al., 2013; Ogrim et al., 2014; Bridwell et al., 2015; Araki et al., 2016; Grane et al., 2016; Zielińska et al., 2016).

In the cued Go-NoGo task a defined cue (S1) indicates that the subsequent stimulus (S2) may require a response. This evokes top-down response preparation processes facilitating speeded reactions (Grane et al., 2016). The CNV is a slow negative potential elicited in the time interval between the cue and the imperative stimulus (S2), and probably indicates response preparation (Ahmadian et al., 2013). The main neural generators of the CNV are thought to be in the frontal cortex (Battaglini et al., 2017) which plays a central role by exerting top-down response preparation (Stuss, 2007). The CNV is considered to be an index of both anticipatory attention for the upcoming stimulus and motor preparation needed to respond (Brunia and Van Boxtel, 2001) and is related to reaction time (RT) and reaction time variability (RTV) (Karalunas et al., 2014). The ERP amplitude generally reflects current neuronal activity (Luck, 2014), and the amplitude of the CNV may therefore represent the neuronal resources involved in the preparatory process. Preparation for fast responding also leads to an augmented need for abortion of the prepared response when the S2-stimulus is a NoGo stimulus. The reactive control processes after S2 therefore include conflict monitoring *and* execution *or* inhibition of the planned response. The N2 is a negative deflection ~200 ms after S2, and is suggested to reflect the cognitive control necessary for interference suppression and successful inhibition (Donkers and Van Boxtel, 2004; Downes et al., 2017) and thereby conflict monitoring or the degree of experienced conflict (Hammerer et al., 2010). The P3, a positive deflection ~300 ms after both stimuli (S1 and S2), has been suggested to indicate the classification of the stimulus and the selection of responses, and in NoGo trials evaluate the inhibitory process after S2 (Aasen and Brunner, 2016). P3 amplitude generally is sensitive to the amount of attentional resources engaged, and will be enhanced if the subject puts more effort into the task, but attenuated if the importance of the stimuli is unclear (e.g., if the given stimulus is target or non-target) or if the task is difficult (Polich, 1987, 2012). The characteristics of the stimuli are therefore essential for the amplitude of P3.

Generally, the literature supports that executive function skills improve in subjects with ASD through childhood and adolescence (Rosenthal et al., 2013), but the maturation is slower and may remain impaired into adulthood. Further, the role of comorbid Attention-Deficit/ Hyperactivity Disorder (ADHD) related to executive dysfunction is also not fully clarified. A recent review of executive functions in ASD, ADHD and comorbid ASD *and* ADHD found inconsistent results across studies attributed to differences in sample characteristics and assessment methods (Craig et al., 2016). They reported response inhibition impaired only in the groups with comorbid ADHD compared to “clean” ASD and typically developing (TD) children. They were not able to identify differences between the diagnostic groups regarding response preparation and monitoring.

Some studies have investigated ERPs associated with attention and inhibition in ASD (Tye et al., 2014; Cui et al., 2016; Faja et al., 2016; Thillay et al., 2016; Kim et al., 2017). Enhanced CNV was reported in children 8–13 years with ASD compared to TD (Tye et al., 2014). Thillay et al. (2016) found enhanced CNV in both ASD and TD before predictable targets, but only in ASD when targets were random. This altered CNV suggests an altered top-down response preparation in ASD. Tye et al. (2014) also reported reduced N2 amplitude enhancement from Go to NoGo trials (the N2-effect) in ASD, but no significant differences in neither N2 Go nor N2 NoGo. Generally larger N2 amplitudes were reported in children 7–11 years with ASD in a flanker test, suggesting that they recruit more neuronal resources when monitoring conflicting information. Kim et al. (2017) found no amplitude differences in N2 Go, N2 NoGo nor N2-effect in kindergartens with ASD. Thus, there are conflicting findings according to N2. A recent meta-analysis of P3 amplitude and latency in ASD (Cui et al., 2016) reported great variability and attribute this to differences in tasks and participants. However, they summarized that ASD showed attenuated P3b amplitudes and attributed this to abnormal information processing in the selection of responses. Deviant Cue P3, N2, and P3 NoGo are frequently found in other neurodevelopmental disorders (Johnstone et al., 2013; Downes et al., 2017).

Age-related changes of ERP components related to response preparation, conflict monitoring, and response inhibition are previously investigated in TD (Tecce, 1971; Cohen, 1973; Jonkman, 2006; Lamm et al., 2006; Lewis et al., 2006; Downes et al., 2017). Jonkman (2006) found CNV amplitude significantly larger in adults than children indicating a linear increase with age. Other studies found a linear increasing CNV in pre-adolescence with maximum amplitude at 15 years (Tecce, 1971; Cohen, 1973). The Cue P3 is also shown to be stronger in children compared to adults. Both the enhanced CNV and Cue P3 are suggesting a higher response preparation (Jonkman, 2006). The amplitude of N2 is typically described as decreasing with age (Jonkman, 2006; Downes et al., 2017), but also dependent on task performance; better performance is associated with reduced amplitude (Lamm et al., 2006). Hammerer et al. (2010) described decreasing N2 from childhood to young adulthood, steeper decreasing in NoGo than Go condition. They suggested this was related to improved executive functions and thus reduced experienced conflict with age. The P3 NoGo is often absent in small children and increase in amplitude until adolescence (Jonkman, 2006). However, to the best of our knowledge, there are few studies of these ERP-components in *adolescents* with ASD.

We have previously reported similar performance between TD and ASD in a visual cued Go-NoGo task (Høyland et al., in review). The task stimuli were split, the first part containing neutral pictures of animals/plants (VCPT) and the second, pictures with emotional faces (ECPT). Degree of social difficulties was determined on all participants by the Social Responsiveness Scales. We found enhanced reaction time in young adolescents correlated with social difficulties, but not the same enhancement in older adolescents. This suggests altered development of emotional understanding in adolescents with ASD. We also found that RTV and social function correlated significantly, but in opposite directions in the two age groups giving a significant interaction between score of social function and age group. In the older adolescents, more social difficulties correlated negatively with RTV. This could indicate better sustained attention in the ASD over 16 years.

The aim of the present study was to identify differences between ASD and TD on ERP- components associated with response preparation, conflict monitoring, and response inhibition during a cued Go-NoGo task. These executive function components may represent cognitive processes relevant for Insistence of Sameness and thereby for the diagnostic category of RRB in ASD. We hypothesized that ERP components associated with response preparation (CNV) were increased in ASD in both Go-NoGo paradigms (VCPT and ECPT). Due to delayed development of executive functions and the clinical feature Insistence of Sameness in ASD, we also expected the conflict monitoring N2-effect to be increased. The components associated with classification of the stimulus and selection of responses (Cue P3 and P3 Go/NoGo) were expected to be unaffected by neutral stimuli (VCPT), but attenuated by emotional stimuli (ECPT) in ASD, due to emotion processing difficulties. Age-related changes in these components between 12 and 21 years were investigated, and we expected more enhanced differences in the young group due to the maturational delay in executive functions in ASD. Lastly, we investigated if RT and RTV was related to the ERP component of response preparation, CNV, and we expected shorter RT and less RTV with increasing CNV amplitude.

## Materials and methods

### Participants

Fifty adolescents with a confirmed diagnosis of ASD without intellectual disability from outpatients attending St. Olavs Hospital, Trondheim, Norway, were included in the study during 2013–2016 (Table 1). The sample consisted of 13 girls and 37 boys, aged 12–21 years, average 15.6 years. The ASD patients were diagnosed according to the ICD-10 F.84 criteria for pervasive developmental disorder based on developmental information and clinical assessments. The Autism Diagnostic Observation Schedule (ADOS) (Lord et al., 2000) was used in 43 of 50 cases.

**Table 1:** Demographics; number (*n*) and mean ± *SD*.

	**TD**		**ASD**	
	***n* = 49**		***n* = 49**	
**GENDER**				
Male	31		36	
Female	18		13	
**ASD SUBGROUP**				
Infantile autism			13	
Asperger disorder			18	
PDD NOS			18	
**AGE–YEARS**				
All	49	15.6 ± 1.8	49	15.6 ± 2.4
<16 years	27	14.3 ± 1.0	26	13.7 ± 1.3
≥16 years	22	17.3 ± 1.1	23	17.8 ± 1.3
**IQ**			49	
Full scale IQ			36	91.9 ± 17.7
Verbal IQ			47	87.6 ± 19.0
Nonverbal IQ			48	98.1 ± 19.3
**SCQ**	47	1.9 ± 2.3	49	18.7 ± 6.7
Infantile autism			13	19.7 ± 6.0
Asperger disorder			18	17.7 ± 6.9
PDD NOS			18	19.0 ± 7.1
**BRIEF**				
**GEC** All	36	42.0 ± 6.0	37	67.6 ± 10.2
<16 years	23	41.9 ± 6.4	22	64.8 ± 8.9
≥16 years	13	42.2 ± 5.4	15	71.6 ± 10.8

*SCQ, Social Communication Questionnaire; BRIEF, Brief Rating Inventory of Executive Function; GEC, Global Executive Composite score; PDD NOS, Pervasive Developmental Disorder Not Otherwise Specified*.

Forty-nine typically developing adolescents, matched for age and gender, were recruited from adjacent schools through invitations/bulletins to all students/parents. In the invitation letter and recruitment posts we invited healthy adolescents. The parents confirmed in writing that their child did not suffer from any chronic disease or psychiatric problems presently or in previously. Eighteen girls and 31 boys between 12 and 20 years were included.

The functioning of networks involved in cognitive control are thought to reach adult level about the age of 15 (Solomon et al., 2014). To test if our results were associated with age we divided the participants into two groups, above and below 16 years of age. The young group included 27 TD and 26 ASD, and the older group included 22 TD and 23 ASD individuals.

Intelligence Quotients, IQs, were obtained for those in the ASD group. Most of the IQs were done previous of this study, including one participant who was assessed using the Leiter test because of specific language problems. The others were tested using the Wechsler tests. Some subjects were tested after recruitment into the current study applying the Wechsler Abbreviated Scales of Intelligence. When the difference between verbal and performance IQs was ≥30, we did not calculate full scale IQ (FIQ). To be included in the study, verbal (VIQ) or performance IQ (PIQ) had to be within the normal variation (≥70). Eighteen (37%) individuals in the ASD group had neuropsychiatric comorbidity, all but one with attention problems [Attention Deficit Disorder (ADD) with or without hyperactivity (ADHD)]. Eight (16%) had more than one comorbid diagnosis. Six (12%) had a diagnosis of epilepsy, all but one with co-occurring ADHD/ADD. Twelve (25%) of the ASD individuals used medication regularly. Four were on stimulants, two used atomoxetine and the six with epilepsy were on antiepileptic medication.

To identify characteristics associated with ASD the parents of all participants completed the lifetime version of the Social Communication Questionnaire (SCQ) (Rutter et al., 2003). The questionnaire is based on the Autism Diagnostic Interview-Revised (ADI-R) (Lord et al., 1994) and is found valid for the ASD diagnosis (Berument et al., 1999; Corsello et al., 2007). It has shown good ability to discriminate between ASD and non-ASD (Chandler et al., 2007). The ASD-group had markedly increased scores on SCQ compared with TD (*p* < 0.001, Table 1).

The parents also filled in the Behavior Rating Inventory for Executive Functioning (BRIEF) (Gioia et al., 2000) as a description of everyday executive function abilities in the participants. BRIEF showed significant differences (*p* < 0.001, Table 1) between ASD and TD.

One of the participants in the ASD group scored >70% on the inattention subscale of the performance test and was excluded. The others, 49 ASD individuals and 49 TD, were included in the study. The behavioral results of the current sample were reported earlier (Høyland et al., in review).

### Experimental task, electrophysiological recording, and analysis

#### Experimental task

We used a cued Go-NoGo task which measures variables of attention and reaction time (Mueller et al., 2010). The categories of visual stimuli (see Figure S1, http://bio-medical.com/products/psytask.html) include 15 pictures of each category; animals, plants and humans in part one (VCPT), and facial emotions (angry, happy, and neutral from Ekmans Pictures of facial affect; Ekman and Friesen, 1976) in part two (ECPT). All participants completed 300 trials VCPT followed by 300 trials ECPT. Each trial consisted of a pair of stimuli (S1–S2). When S1 was a cue (animal/angry face), the S2 was either animal/angry face (Go trials), or plant/happy face (NoGo trials). When S1 was plant/happy face they should never give response to S2 (ignore trials). S1 and S2 are presented for 100 ms with an 1,100 ms inter-stimulus interval and an inter-trial interval of 3,000 ms. The trials are grouped into blocks separated by a short break. In each block, a unique set of five pictures from each picture category are selected. Each block consists of a pseudo-random presentation of 100 stimulus pairs with equal probability for each trial category. The participants were told to response by pressing a button with their index finger as quickly as possible without making mistakes in all Go trials and otherwise refrain from responding. For more details, see also Høyland et al. (in review).

During the task, subjects were seated in a comfortable chair that was 1.2 m from the computer screen. The pictures (size ~20 × 15 cm) were presented in the middle of an 18-inch monitor using the Psytask (http://bio-medical.com/products/psytask.html) software (from Bio-medical, Clinton Township, Michigan USA). The time interval from the presentation of the second stimulus to the response (RT) and RTV was registered by VCPT/ECPT software. The ERPs are averaged through trials with correct responses. The software also registered omissions and commissions.

#### Electrophysiological recordings

Electroencephalogram (EEG) was recorded using a Mitsar (http://www.mitsar-medical.com) EEG system with a 19-channel tin electrode cap (Electro-cap International, Eaton, OH, USA). The electrodes were placed according to the international 10-20-system. The input signals were referenced to earlobe electrodes and filtered between 0.5 and 50 Hz and digitized at a sampling rate of 500 Hz. Impedance was kept below 5 kΩ for all electrodes. Quantitative data were obtained from the WinEEG software (www.mitsar-medical.com) in common average montage prior to data processing. Eye blink artifacts were corrected by zeroing the activation curves of individual independent components corresponding to eye blinks. In addition, epochs of the filtered EEG with excessive amplitude (>100 μV) and/or slow (>50 μV in the 0–1 Hz-band) and excessive fast (>35 μV in the 20–35 Hz-band) frequency activity were automatically excluded from further analysis.

All participants had a 6-min resting EEG registration and a specialist in clinical neurophysiology examined the registrations and found no epileptic activity.

**Figure 1:**
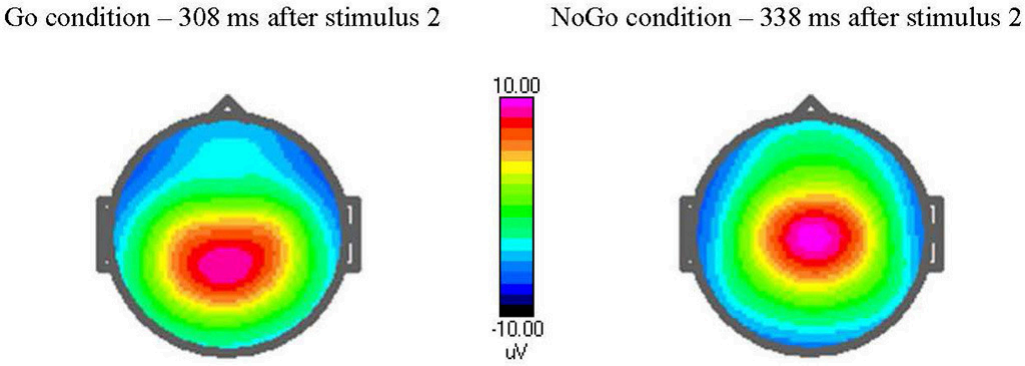
Amplitude voltage map [in μvolts (μV)] at peak latency in Go and NoGo trials. The cued Go NoGo task elicits a maximum Go P3-component after 308 ms when stimulus 2 is target (Go condition) and a NoGo P3-component after 338 ms when stimulus 2 is non-target. Observe the topography of the P3, with a maximum at more parietal site in Go condition and centrally in NoGo condition.

The ERPs for each individual were based on averaging the trials of the respective task condition with correct response after artifact correction. The number of artifact-free trials averaged were 269 (±22.4, range 191–300) for TD, 261(±37.9, range 109–295) for ASD. This makes a non-significant difference in averaged trials. The ERPs were measured by convention as mean or peak amplitudes in the stated electrode and time window as showed by the grand average file, see Table 2. The topography of the P3 components is illustrated in Figure 1.

**Table 2:** Electrophysiological measures, ERPs.

Cue P3	Maximum positive peak in Pz 260–360 ms after S1
CNV	Averaged amplitude in Cz 1,000–1,100 ms after S1(immediately before S2)
N2 Go/ NoGo	Maximum negative amplitude in Fz 90–290 ms after stimuli 2
P3 Go	Maximum positive peak in Pz 260–360 ms after S2
P3 NoGo	Maximum positive peak in Cz 270–420 ms after S2

### Study design and outcomes

The primary outcome for the current study was to compare differences between TD and ASD in the amplitude of the following ERPs elicited during a cued Go-NoGo task: Cue P3, CNV, N2 Go and NoGo, P3 Go and P3 NoGo. The N2-effect was also calculated as N2 Go minus N2 NoGo. Outcomes were analyzed for the whole group of participants, and separately within each of the two age groups.

### Statistical analysis

The descriptives for all ERPs are reported. Subsequently, ERP amplitudes were analyzed as dependent variables in mixed model analyses with subject as random effect, and ECPT vs. VCPT, gender, age group, and diagnosis (ASD vs. TD) as independent variables. We did the analyses first for the whole sample, then separately for the two age groups. Finally, we also included the interaction between diagnosis and age group as independent variable. We repeated the analyses for ASD without comorbid ADHD vs. TD. We also made a scatter-plot (Loess curve) with CNV in both ECPT and VCPT as function of age. Partial correlation with gender as covariate was used to explore the relationship between the performance measures RT/RTV and CNV. All analyses were adjusted for gender.

Normality of residuals was checked by visual inspection of Q-Q plots. Statistical analyses were carried out in IBM SPSS Statistics 23.0. Two-sided *p* < 0.05 were considered statistically significant, however, due to multiple comparisons *p*-values between 0.01 and 0.05 should be interpreted with caution.

### Ethics

The study was approved by the Norwegian Regional Committee for Medical and Health Research Ethics South East (2013/1236/REK South-East). Written informed consent was obtained from participants and/or parents when necessary due to age.

## Results

### Total sample

**Figure 2:**
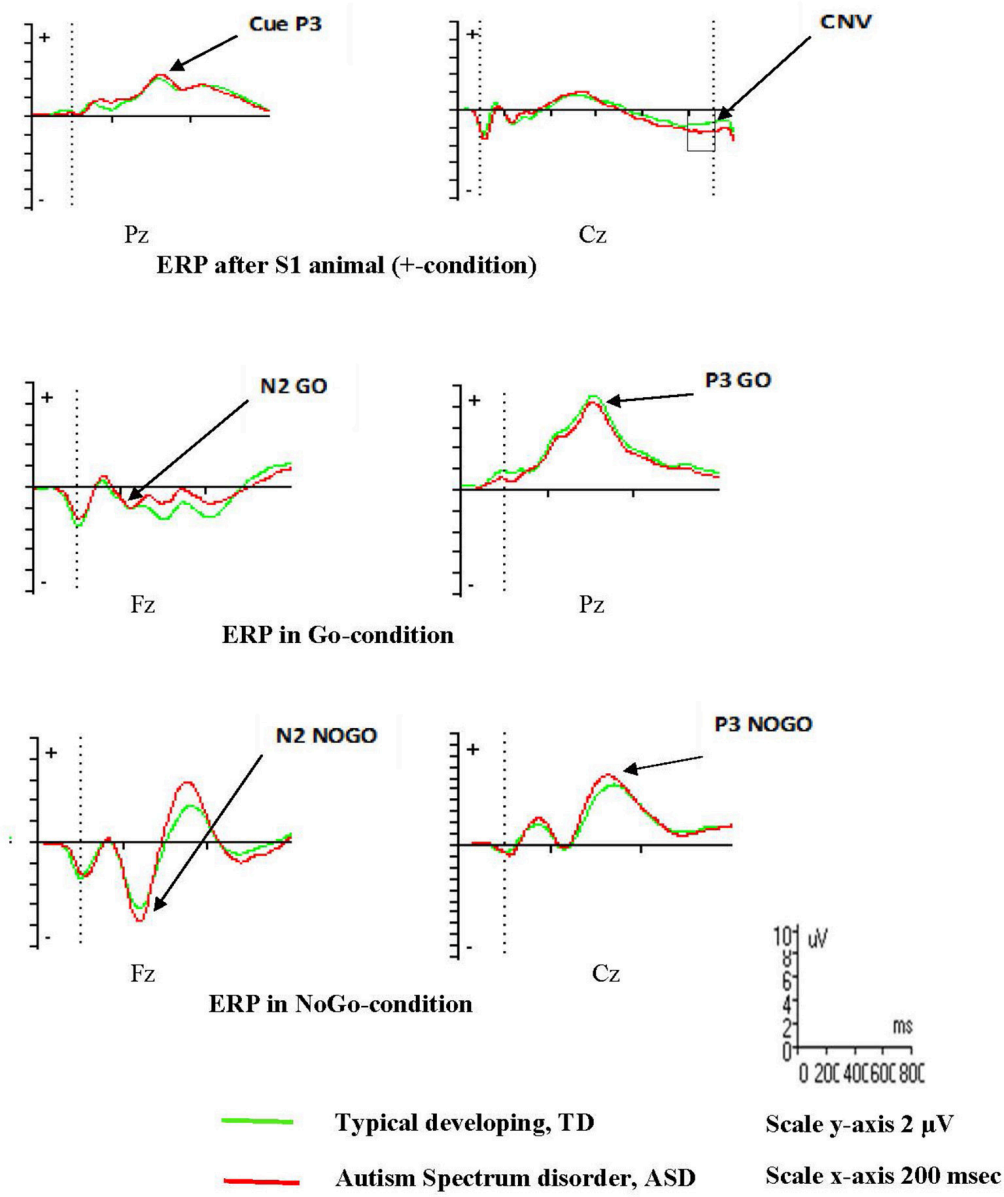
Event Related Potentials, ERPs, VCPT adolescents 16 years and older.

ERPs in the three midline electrodes (Fz, Cz, Pz) are presented in supplement (Figures S2, S3), and examples are provided in Figure 2. Descriptives of ERP amplitudes in the different groups are shown in Table 3. None of the ERPs associated with response preparation (Cue P3 and CNV) and conflict monitoring and response inhibition (N2 Go/ NoGo, N2-effect, P3 Go/NoGo) were significantly different between the ASD and TD groups in the combined VCPT and ECPT (Table 4).

**Table 3:** Event Related Potentials, ERPs, in VCPT and ECPT, for TD, ASD, and ASD without comorbid ADHD (ASD–ADHD).

		**VCPT**			**ECPT**		
		***n* = 49 TD**	***n* = 49 ASD**	***n* = 32 ASD–ADHD**	***n* = 49 TD**	***n* = 49 ASD**	***n* = 32 ASD–ADHD**
CNV	All	−1.86 ± 1.2	−1.96 ± 1.5	−2.05 ± 1.6	−2.12 ± 1.4	−2.20 ± 1.8	−2.33 ± 1.73
	<16 years	−2.08 ± 1.3	−1.53 ± 1.6	−1.33 ± 1.6	−2.24 ± 1.4	−1.74 ± 1.9	−1.56 ± 2.0
	≥16 years	−1.59 ± 1.0	−2.43 ± 1.2	−2.59 ± 1.2	−1.97 ± 1.3	−2.72 ± 1.5	−2.90 ± 1.3
N2 Go	All	−4.97 ± 2.7	−4.07 ± 2.9	−4.42 ± 2.8	−2.96 ± 2.3	−2.79 ± 3.2	−3.61 ± 3.1
	<16 years	−5.90 ± 2.0	−4.63 ± 3.1	−5.55 ± 2.6	−3.57 ± 2.0	−3.45 ± 3.3	−5.11 ± 2.5
	≥16 years	−3.83 ± 3.0	−3.43 ± 2.6	−3.43 ± 2.4	−2.21 ± 2.5	−2.05 ± 2.9	−2.29 ± 3.1
N2 NoGo	All	−9.13 ± 3.3	−8.74 ± 3.9	−9.86 ± 3.3	−4.98 ± 2.6	−5.21 ± 4.0	−6.52 ± 4.1
	<16 years	−10.57 ± 2.7	−8.85 ± 4.7	−10.98 ± 3.9	−5.15 ± 2.6	−5.24 ± 4.9	−7.63 ± 4.8
	≥16 years	−7.35 ± 3.3	−8.60 ± 2.9	−8.88 ± 2.3	−4.77 ± 2.6	−5.17 ± 2.9	−5.54 ± 3.1
N2-effect ^a^	All	4.15 ± 2.6	4.67 ± 3.2	5.44 ± 2.8	2.02 ± 2.2	2.42 ± 3.1	2.90 ± 3.5
	<16 years	4.67 ± 2.9	4.23 ± 3.0	5.43 ± 2.6	1.58 ± 1.9	1.80 ± 3.3	2.51 ± 4.0
	≥16 years	3.52 ± 2.1	5.17 ± 3.3	5.44 ± 3.1	2.56 ± 2.4	3.13 ± 2.7	3.24 ± 3.1
Cue P3	All	5.46 ± 2.7	5.66 ± 3.0	5.27 ± 2.4	4.56 ± 2.2	5.26 ± 2.9	5.05 ± 2.08
	<16 years	6.09 ± 2.4	5.92 ± 3.1	5.55 ± 2.8	5.05 ± 2.2	5.87 ± 3.1	6.03 ± 3.1
	≥16 years	4.69 ± 2.8	5.37 ± 2.9	5.03 ± 2.0	3.97 ± 2.1	4.56 ± 2.5	4.19 ± 2.3
P3 Go	All	9.24 ± 4.1	9.36 ± 3.0	9.60 ± 2.9	8.24 ± 2.9	8.80 ± 4.3	9.47 ± 4.6
	<16 years	9.21 ± 5.0	9.64 ± 3.1	9.75 ± 3.6	8.57 ± 3.1	8.96 ± 4.4	9.44 ± 5.2
	≥16 years	9.27 ± 2.8	9.06 ± 2.9	9.46 ± 2.3	7.83 ± 2.5	8.59 ± 4.3	9.50 ± 4.3
P3 NoGo	All	11.66 ± 4.2	11.94 ± 6.0	13.13 ± 6.7	10.57 ± 4.5	10.08 ± 6.7	11.10 ± 7.41
	<16 years	11.24 ± 3.5	10.76 ± 6.4	11.88 ± 8.1	9.60 ± 3.0	8.48 ± 6.9	9.07 ± 8.7
	≥16 years	12.17 ± 4.9	13.27 ± 5.3	14.23 ± 5.3	11.77 ± 5.7	11.88 ± 6.1	12.89 ± 5.8

a *N2−effect, N2 Go vs. N2 NoGo. All amplitudes reported in μV, mean ± SD*.

**Table 4:** Mixed model analysis with the reported Event Related Potentials, ERPs, as dependent variables.

		**ASD vs. TD**	**(ASD–ADHD) vs. TD**
		**β (confidence−interval), *p***	**β (confidence−interval), *p***
CNV	All	−0.08 (−0.63 to 0.48), *p* = 0.79	−0.15 (−0.78 to 0.48), *p* = 0.63
	<16 years	0.56 (−0.25 to 1.36), *p* = 0.17	0.75 (−0.23 to 1.74), *p* = 0.13
	≥16 years	−0.86 (−1.55 to −0.18), *p* = 0.015^*^	−1.01 (−1.74 to −0.28), *p* = 0.008^**^
	Interaction with age group	*p* = 0.017^*^	*p* = 0.006^**^
N2 Go	All	0.53 (−0.44 to 1.50), *p* = 0.28	−0.29 (−1.30 to 0.72), *p* = 0.57
	<16 years	0.73 (−0.56 to 2.03), *p* = 0.26	−0.57 (−1.83 to 0.70), *p* = 0.37
	≥16 years	0.26 (−1.27 to 1.80), *p* = 0.73	0.04 (−1.61 to 1.69), *p* = 0.96
	Interaction with age group	*p* = 0.68	*p* = 0.44
N2 NoGo	All	0.07 (−1.18 to 1.32), *p* = 0.91	−1.25 (−2.71 to −0.28), *p* = 0.016^*^
	<16 years	0.89 (−0.99 to 2.76), *p* = 0.35	−1.45 (−3.34 to 0.44), *p* = 0.13
	≥16 years	−0.98 (−2.57 to 0.60), *p* = 0.22	−1.43 (−3.01 to 0.15), *p* = 0.075
	Interaction with age group	*p* = 0.20	*p* = 0.76
N2−effect^a^	All	0.46 (−0.47 to 1.39), *p* = 0.33	1.21 (0.17 to 2.24), *p* = 0.023^*^
	<16 years	−0.15 (−0.39 to 1.08), *p* = 0.80	0.88 (−0.58 to 2.34), *p* = 0.23
	≥16 years	1.24 (−0.18 to 2.67), *p* = 0.085	1.47 (0.07 to 3.01), *p* = 0.060
	Interaction with age group	*p* = 0.19	*p* = 0.69
Cue P3	All	0.43 (−0.56 to 1.42), *p* = 0.39	0.23 (−0.80 to 1.26), *p* = 0.66
	<16 years	0.22 (−1.15 to 1.59), *p* = 0.75	−0.11 (−1.69 to 1.47), *p* = 0.89
	≥16 years	0.78 (−0.63 to 2.19), *p* = 0.27	0.42 (−0.92 to 1.76), *p* = 0.53
	Interaction with age group	*p* = 0.78	*p* = 0.95
P3 Go	All	0.34 (−0.98 to 1.67), *p* = 0.61	0.92 (−0.62 to 2.46), *p* = 0.24
	<16 years	0.32 (−1.63 to 2.28), *p* = 0.74	0.43 (−2.15 to 3.02), *p* = 0.74
	≥16 years	0.45 (−1.30 to 2.22), *p* = 0.60	1.21 (−0.51 to 2.93), *p* = 0.16
	Interaction with age group	*p* = 0.92	*p* = 0.90
P3 NoGo	All	−0.08 (−2.16 to 1.99), *p* = 0.99	1.11 (−1.35 to 3.57), *p* = 0.37
	<16 years	−0.95 (−3.68 to 1.77), *p* = 0.49	−0.27 (−3.95 to 3.41), *p* = 0.88
	≥16 years	1.16 (−1.92 to 4.25), *p* = 0.45	2.16 (−1.15 to 5.47), *p* = 0.20
	Interaction with age group	*p* = 0.49	*p* = 0.56

a *N2-effect, N2 Go vs. N2 NoGo*.

* Significant at the 0.05 level (2-tailed).

** *Significant at the 0.01 level (2-tailed). The fixed effects were diagnostic group [typically developing (TD) vs. autism spectrum disorder (ASD)], task (VCPT vs. ECPT) and gender. We first analyzed using the whole sample, then separately for each age group. We then included the interaction between age group and diagnostic group. The analyses were finally recomputed for the groups TD vs. ASD without comorbid ADHD (ASD–ADHD)*.

Seventeen of the adolescents in the ASD group had comorbid ADHD. When excluding the ASD with comorbid ADHD, the N2 NoGo was significantly increased in the ASD group (*p* = 0.016, Table 4). The N2-effect was correspondingly enhanced (*p* = 0.023, Table 4).

We found significant correlations between RT and CNV (VCPT *r* = 0.29, *p* = 0.004; and ECPT *r* = 0.47, *p* < 0.001) for all participants. We also found significant correlations between RTV and CNV (VCPT *r* = 0.29, *p* = 0.004*;* and ECPT *r* = 0.34, *p* = 0.001).

### Age related differences

**Figure 3:**
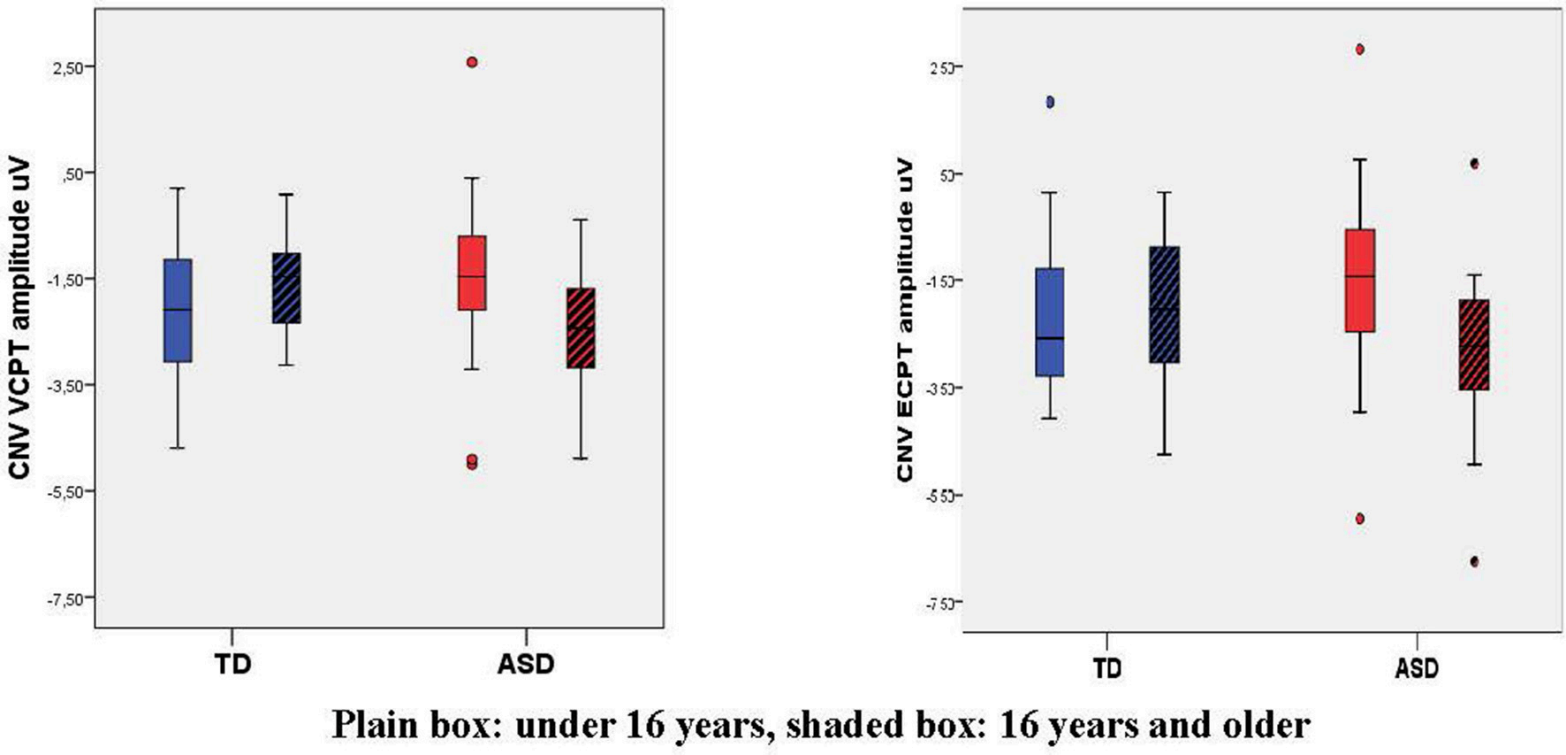
Box plot of Contingent Negative Variation, CNV, in VCPT/ECPT. Mean CNV VCPT and ECPT increases from young to old in TD, decreases in ASD, giving a significant age-group × diagnosis interaction.

**Figure 4:**
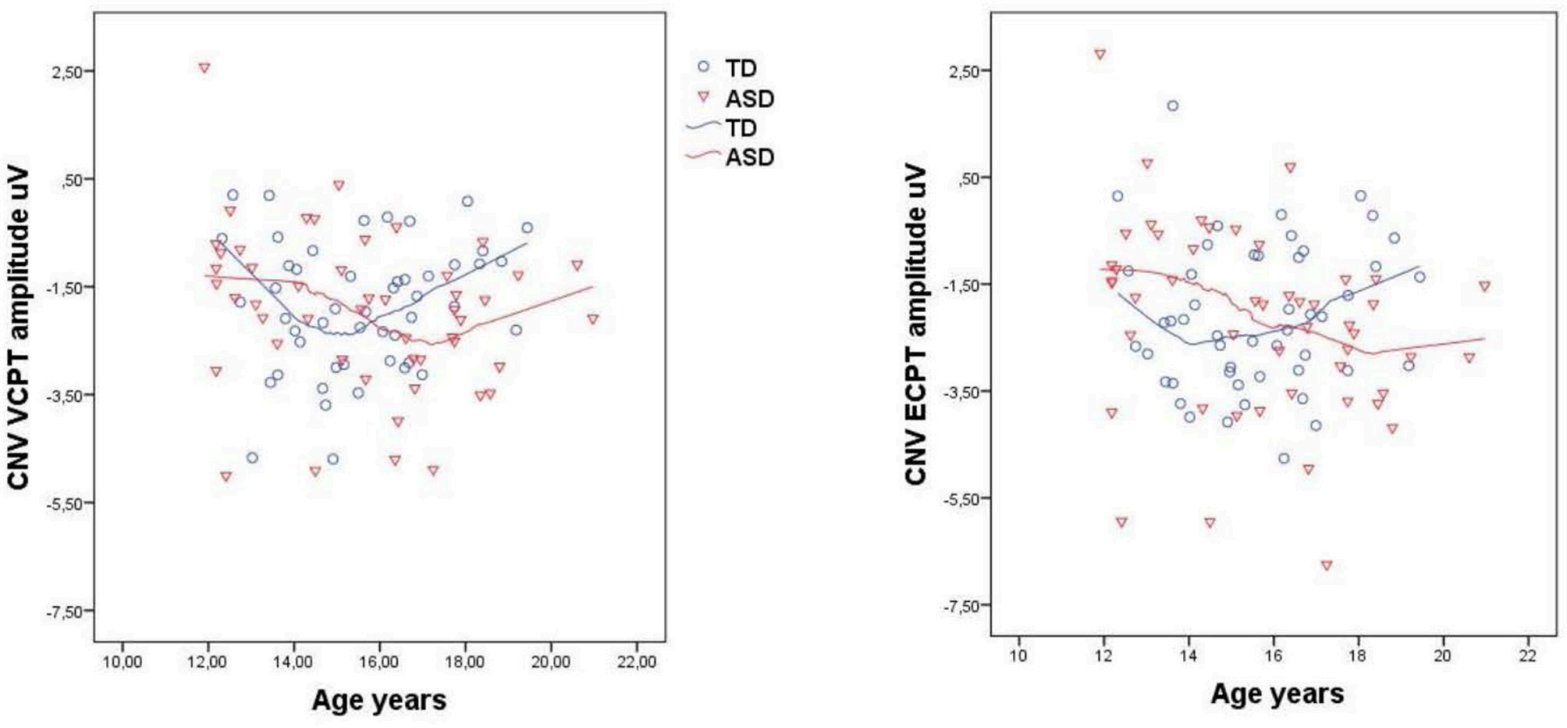
Contingent negative variation, CNV, in VCPT/ECPT vs. age in typical developing adolescents (TD) and adolescents with autism spectrum disorders (ASD). Scatterplots with loess curves fitted to each diagnostic group.

There were no significant differences in the CNV amplitudes in the combined VCPT and ECPT data between cases and controls in the young age group, see Table 4. In the older age group, CNV was significantly (*p* = 0.015) enhanced in ASD compared to TD (Table 4). We also found a corresponding age × diagnosis interaction for CNV (Table 4, Figure 3). When plotting CNV vs. age as a continuous scale we found maximum amplitude at ~15 years in TD, 17 years in ASD (Figure 4). The other ERPs recorded were not significantly different between ASD and TD in either age group (Table 4).

Repeating the analyses after excluding the participants with comorbid ADHD generally increased the differences between ASD and TD (Table 4).

## Discussion

We show ERPs related to response preparation, conflict monitoring and response inhibition in adolescents with ASD, all related to the clinical phenomenon Insistence of Sameness. The main findings of the present study were age related alterations in CNV and differences in N2 in the visual cued Go-NoGo task in ASD, while the behavioral performance was similar to the TD group. The age-related development of CNV in adolescents with ASD is not described previously. Our results contribute to the neurophysiology associated with executive dysfunction in ASD, and suggest biological underpinnings associated to a core RRB characteristic in ASD.

An enhanced CNV is thought to reflect increased response preparation (Brunia and Van Boxtel, 2001). This may lead to reduced flexibility and thus problems with set-shifting; features related to Insistence of Sameness (Yerys et al., 2009). Further it may be linked to the “sticky fixation” phenomenon associated to reduced selective inhibition described by Holmboe et al. (2010). Mosconi et al. (2009) also relate reduced inhibitory control to the Insistence of Sameness category of RRB. *Attenuated* CNV is reported to be associated with attentional problems in ADHD (Doehnert et al., 2010). The present findings of increased CNV in ASD above 16 years of age may represent a superior detail-focused cognitive style (Happe and Frith, 2006), the opposite of attention deficits. The detail-focused style is part of the altered perception in autism (Mottron et al., 2009), which may be associated with Insistence of Sameness. The CNV was significantly correlated to both RT and RTV for all participants (increased CNV associated with reduced reaction time and less RTV) in line with previous reports (Karalunas et al., 2014). This relation may reflect the neuronal resources involved in the preparatory process and thus performance. We did not replicate earlier findings of enhanced CNV in younger children with ASD (Tye et al. (2014). This may be due to differences in age of participants, paradigms, inter-trial interval and also time interval for assessing the CNV. In a longitudinal study, Doehnert et al. (2010) found reduced CNV in ADHD compared to TD from childhood to adolescence. After excluding participants with comorbid ADHD, we found an increased CNV in the group over 16 years, showing the same effect of ADHD. Thus, the reported enhanced CNV seem to be specific for ASD and may indicate a pathophysiological mechanism of executive dysfunction in ASD which could be overlapping with RRB.

The current findings of an ASD specific age-related development of CNV in adolescence are in line with abnormal brain development in ASD (Solomon et al., 2014). Earlier studies have found increasing CNV amplitude in TD until 15 years and thereafter gradual attenuation (Tecce, 1971; Cohen, 1973). Our findings are in line with this observation. In the ASD group, however, the CNV amplitude increased until 17 years before attenuating. This suggests an altered development of neurophysiological processes underlying CNV in ASD. At the age of 20 years, the upper age-limit in our study, the CNV amplitude remained enhanced in the ASD group, suggesting that these abnormalities may persist into adulthood (Thillay et al., 2016). However, longitudinal studies are needed to determine the life span patterns of neurophysiological parameters in ASD.

We found no difference between the total ASD group and TD in the amplitude of N2 NoGo or N2-effect. Tye et al. (2014) reported attenuated N2-effect in children with ASD aged 8–13 years. Faja et al. (2016) found overall enhanced N2-components in ASD in children aged 7–11 years, but similar N2-effect. Both these studies included children younger than our participants. Several studies found decreasing N2 NoGo from childhood to adulthood in TD (Lamm et al., 2006; Hammerer et al., 2010). A reduced N2-effect is reported in ADHD (Albrecht et al., 2008). The ASD group in the study by Faja et al. (2016) included 8 children (29%) with ADHD which may affect their results. In our study, we included 17 ASD participants with comorbid ADHD. When they were excluded, we found both N2 NoGo and N2-effect significantly enhanced. These results are in line with our hypothesis, but must be interpreted with caution considering the multiple statistical testing. Thus, both the age and the inclusion/ exclusion of participants with comorbid ADHD may influence the results. N2 is supposed to represent conflict monitoring (Donkers and Van Boxtel, 2004) which subsequently may be related to experienced conflict (Hammerer et al., 2010). Thus, these findings of N2-deviance may also be related to the clinical feature of Insistence of Sameness. Taken together, the current findings of both CNV and N2-deviance in ASD seem to implicate pathological neuronal excitability as a link between executive function and Insistence of Sameness.

We found similar amplitudes in the ASD and TD groups in the P3 components. In a recent meta-analysis of ASD compared to TD, Cui et al. (2016) found diverging P3 results which they attributed to high heterogeneity among the studies. They reported some evidence for reduced P3b amplitude in ASD. We did not find significant attenuation of P3 in ASD. This discrepancy could be due to differences in participants and paradigms (Cui et al., 2016). The performance-results in our study were mainly similar between TD and ASD supporting normal abilities in classification of the stimulus and selection of responses after S2.

An interesting aspect of the current findings is the relations between VCPT and ECPT. The ASD had basically equivalent ERPs to TD despite the emotional content of the stimuli. Thus, we did not confirm our hypothesis of a deviant effect of emotional pictures in ASD, and our results are in line with previous findings; participants with ASD are able to recognize basic emotions (Tanaka et al., 2012). Generally, the ERPs related to target classification (Cue P3/P3) of the emotional stimuli in the ECPT were corresponding to the ERPs in VCPT, but attenuated. Also, the age-related changes in CNV in the ASD group appeared both in VCPT and ECPT. In N2 Go and N2 NoGo the attenuation from VCPT to ECPT is significant in both TD and ASD. This may represent influence of emotional stimuli on attention and information-processing (Delplanque et al., 2006; Conroy and Polich, 2007). Since the VCPT was always presented before the ECPT, the lack of difference may also reflect exhaustion of the participants.

### Strengths and limitations of the study

We included patients previously diagnosed with ASD, but did not repeat the diagnostic assessment. The distribution between the diagnostic subgroups shows an overrepresentation of PPD-NOS in the participants under the age of 16 years. However, there were no significant differences in the ASD symptoms as assessed by SCQ. We did not perform tests to estimate IQs for TD, but the parents of our control group reported no learning problems or psychiatric problems, and they were recruited from school children with normal school performance. Individuals with classical autism typically have significantly lower verbal IQs compared to performance IQs, although this varies within the ASD group. This situation also makes it challenging to match a control group (Harms et al., 2010).

We used the BRIEF, a parent-report measure, as a description of the presence of executive dysfunction in the participants. Research indicates that disagreement exists between performance-based tests and parent-report measures of executive functions (Silver, 2014). Performance-based measurements of executive functions could have contributed to a broader evaluation of executive dysfunction in the participants. We also used parent-report BRIEF for all participants even though some of them were over 18 years old. This because we had information that all participants still lived with their parents and we wanted to use the same BRIEF method across age groups.

The participants were in the ECPT asked to recognize a single basic emotion, anger. They also implicitly had to exclude happy as an emotion to define the “Go condition.” Previous studies have shown that more complicated and subtle emotional expressions are more challenging to recognize for individuals with ASD than the basic emotions (Behrmann et al., 2006). Thus, the present paradigm may have reduced the opportunity to find significant differences in our study, leading to Type II error. All participants were tested by the same technician in the same lab to reduce variations caused by testing conditions.

## Conclusion

The current study of ERPs during a cued Go-NoGo task indicates age-dependent alterations of CNV (related to response preparation), and N2 (related to conflict monitoring) in ASD. These neurophysiological abnormalities during an executive function task may be related to Insistence of Sameness, a core clinical feature in ASD. Our results also underscore the importance of controlling for ADHD comorbidity when interpreting ERPs in an ASD sample. The pathophysiological underpinnings of executive dysfunction in ASD should be further investigated to learn more of how this phenomenon is related to core characteristics of ASD.

## Author contributions

AH, GØ, TN, and OA contributed to the design of the work, the analyses and interpretation of data. SL, SH, and TT contributed to interpretation of the data. AH and ME carried out EEG-analyses and contributed to interpretation of the data. All authors drafted the manuscript and revised it and gave their final approval of the version to be published. All authors also agreed to be accountable for the work in ensuring that questions related to the accuracy or integrity of any part of the work were appropriately investigated and resolved.

### Conflict of interest statement

The authors declare that the research was conducted in the absence of any commercial or financial relationships that could be construed as a potential conflict of interest.
