# Supplementary material for: Event-Related Potentials in a Cued Go-NoGo Task Associated with Executive Functions in Adolescents with Autism Spectrum Disorder; A Case-Control Study
Source: Front Neurosci. 2017 Jul 11;11:393. doi: 10.3389/fnins.2017.00393 (PMC5504259; doi:10.3389/fnins.2017.00393)

Figure S2. Event Related Potentials, ERPs, from midline electrodes from participants under 16 years of age

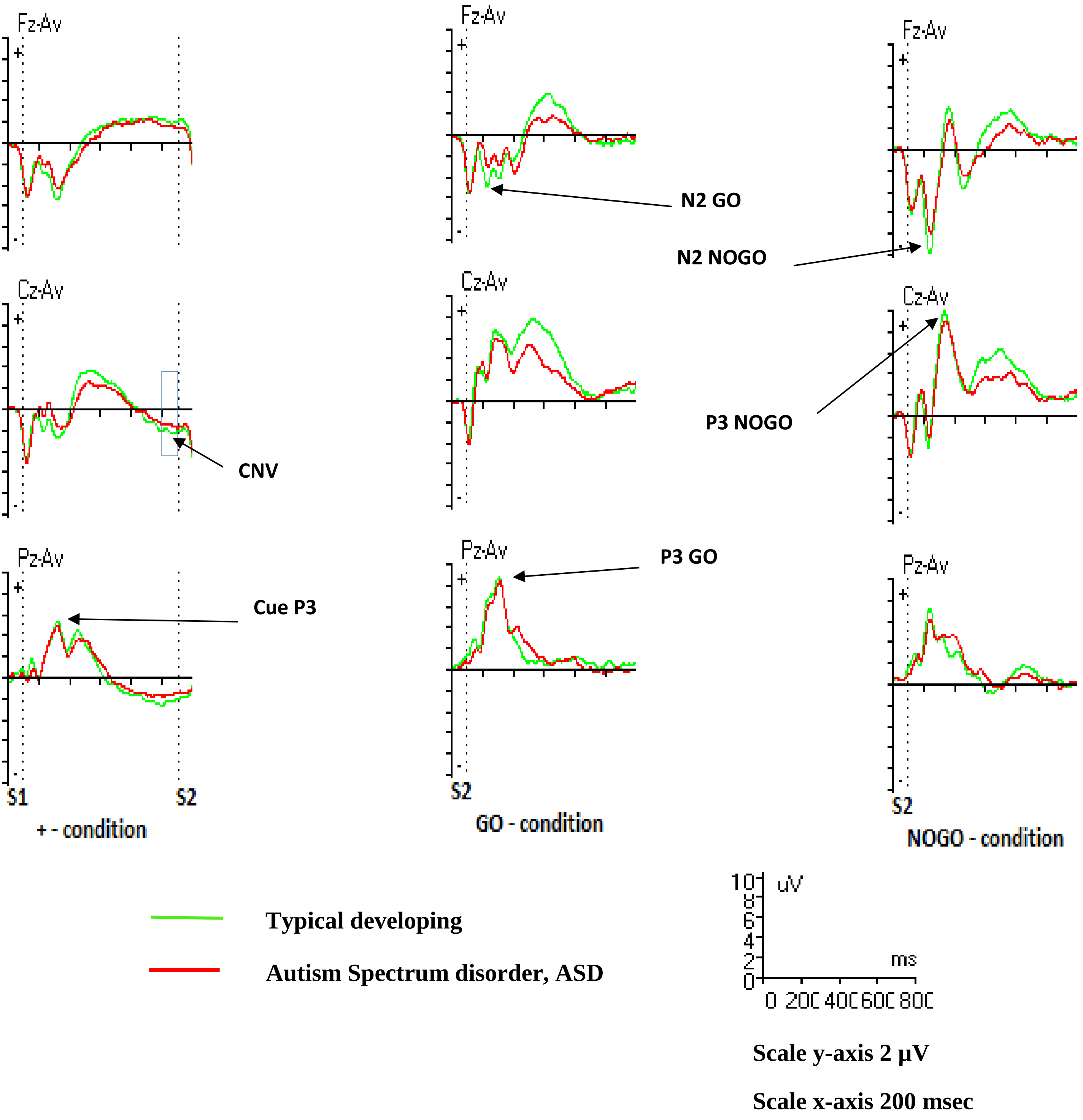

Supplement: Supplementary file 2 [file DataSheet2.PDF]
