# Supplementary material for: Event-Related Potentials in a Cued Go-NoGo Task Associated with Executive Functions in Adolescents with Autism Spectrum Disorder; A Case-Control Study
Source: Front Neurosci. 2017 Jul 11;11:393. doi: 10.3389/fnins.2017.00393 (PMC5504259; doi:10.3389/fnins.2017.00393)
Supplement: Supplementary file 3 [file DataSheet3.PDF]

Figure S3. Event Related Potentials, ERPs, from midline electrodes from participants 16 years of age and older

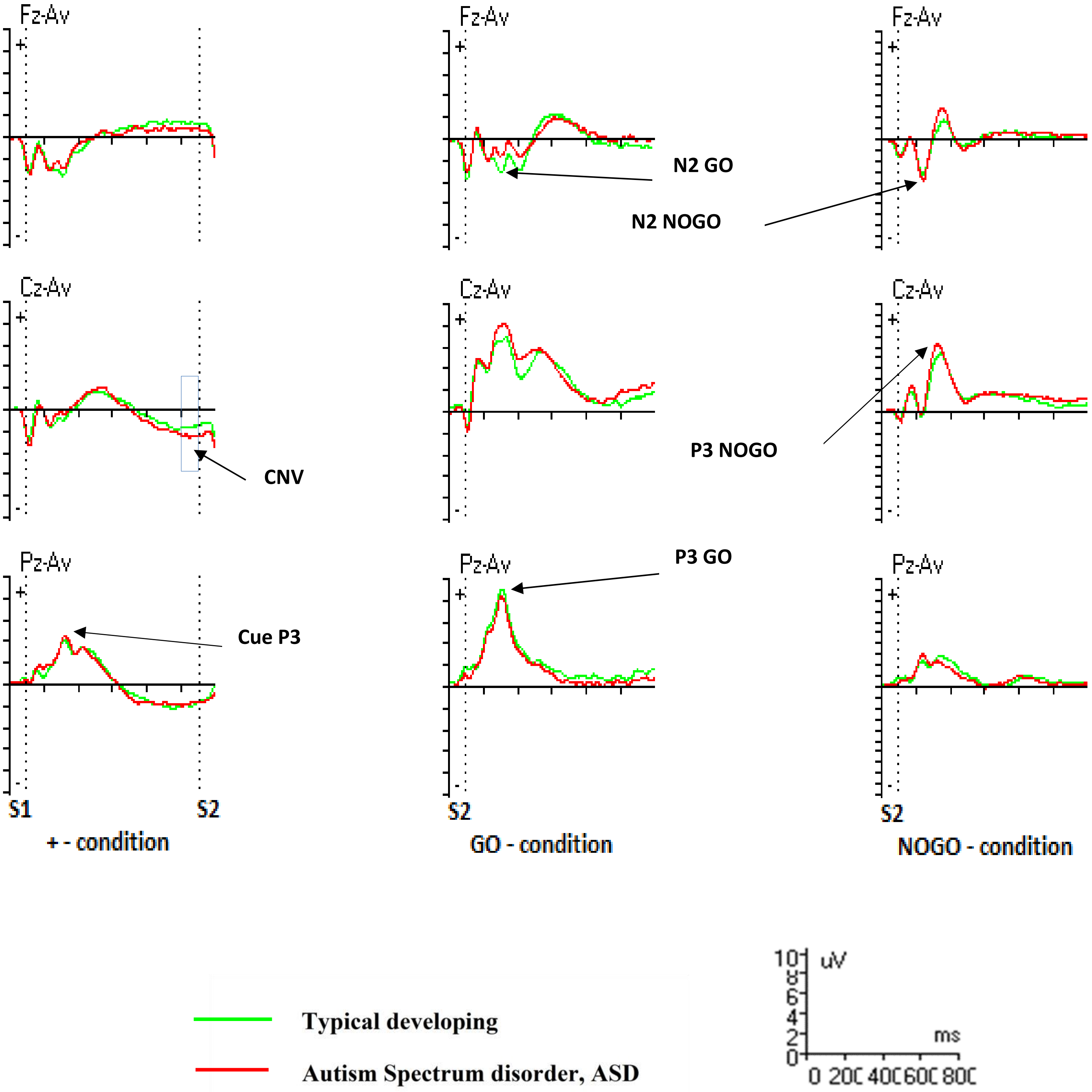

Scale y-axis 2 µV

Scale x-axis 200 msec
